# Extent of white matter lesion is associated with early hemorrhagic transformation in acute ischemic stroke related to atrial fibrillation

**DOI:** 10.1002/brb3.2250

**Published:** 2021-06-14

**Authors:** Lucio D'Anna, Filippos T. Filippidis, Kirsten Harvey, Marilena Marinescu, Paul Bentley, Eleni Korompoki, Roland Veltkamp

**Affiliations:** ^1^ Department of Stroke and Neuroscience Charing Cross Hospital, Imperial College London NHS Healthcare Trust London UK; ^2^ Department of Brain Sciences Imperial College London London UK; ^3^ Department of Primary Care and Public Health, School of Public Health Imperial College London London UK; ^4^ Department of Neurology Alfried‐Krupp Krankenhaus Essen Germany; ^5^ Department of Neurology University Hospital Heidelberg Heidelberg Germany

**Keywords:** acute ischemic stroke, atrial fibrillation, Fazekas score, hemorrhagic transformation

## Abstract

**Background:**

Hemorrhagic transformation (HT) after stroke, related to atrial fibrillation (AF), is a frequent complication, and it can be associated with a delay in the (re‐)initiation of oral anticoagulation therapy. We investigated the effect of the presence and severity of white matter disease (WMD) on early HT after stroke related to AF.

**Methods:**

A consecutive series of patients with recent (<4 weeks) ischemic stroke and AF, treated at the Hyper Acute Stroke Unit of the Imperial College London between 2010 and 2017, were enrolled. Patients with brain MRI performed 24–72 h from stroke onset and not yet started on anticoagulant treatment were included. WMD was graded using the Fazekas score.

**Results:**

Among the 441 patients eligible for the analysis, 91 (20.6%) had any HT. Patients with and without HT showed similar clinical characteristics. Patients with HT had a larger diffusion‐weighted imaging (DWI) infarct volume compared to patients without HT (*p* < .001) and significant difference in the distribution of the Fazekas score (*p* = .001). On multivariable analysis, HT was independently associated with increasing DWI infarct volume (odd ratio (OR), 1.03; 95% confidence interval (CI), 1.01–1.05; *p* < .001), higher Fazekas scores (OR, 1.94; 95% CI, 1.47–2.57; *p* < .001) and history of previous intracranial hemorrhage (OR, 4.80; 95% CI, 1.11–20.80; *p* = .036).

**Conclusions:**

Presence and severity of WMD is associated with increased risk of development of early HT in patients with stroke and AF. Further evidence is needed to provide reliable radiological predictors of the risk of HT in cardioembolic stroke.

## Graphical abstract

The presence and severity of white matter disease is associated with an increased risk of development of early haemorrhagic transformation in patients with stroke and atrial fibrillation

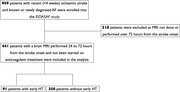

## 1 INTRODUCTION

Early hemorrhagic transformation (HT) of the infarct occurs in 5.9% (Valentino et al., 2017) to 50.81% (Wei et. et al., 2019) of patients with acute ischemic stroke. The risk of HT is influenced by age (Marsh et al., 2013), presence of large infarct (Kablau et al., 2011; Muscari et al., 2020; Paciaroni et al., 2018), use of anticoagulants before the stroke (Pande et al., 2020), severe neurologic deficit (Kidwell et al., 2002), congestive heart failure (Paciaroni et al., 2018), hyperglycemia (Muscari et al., 2020), renal impairment (Marsh et al., 2013), low platelet count (Prodan et al., 2010), elevated systolic blood pressure (Álvarez‐Sabín et al., 2013) and use of reperfusion therapies (Zhang et al., 2014). While the majority of the HTs are asymptomatic and believed to be innocuous in some studies (Annan et al., 2015; Berger et al., 2001; Libman et al., 2005), HT was associated with adverse clinical outcomes such as mortality and disability in other studies (Dzialowski et al., 2007; Paciaroni et al., 2018, 2008; Park et al., 2012). Notably, the presence of HT is associated with a delay in the initiation of oral anticoagulation therapy after stroke, in patients with atrial fibrillation (AF), which may increase the risk of early stroke recurrence (Paciaroni et al., 2018). A better understanding of the factors underlying HT may aid the early identification of patients at high risk of HT leading to an earlier and safer initiation of anticoagulation therapy in selected patients (Paciaroni et al., 2016).

So far, most of the studies on early HT after acute ischemic stroke did not control for the effect of white matter disease (WMD) (He et al., 2019; Kerenyi et al., 2006; Kunte et al., 2012; Marsh et al., 2013, 2016; Muscari et al., 2020; Paciaroni et al., 2008; Tan et al., 2014; Valentino et al., 2017). Moreover, the effect has been investigated in cohorts with mixed stroke etiologies and with a focus on thrombolysis (Bivard et al., 2016; El Nawar et al., 2019; Fierini et al., 2017; Kongbunkiat et al., 2017). Specifically, the association between WMD severity and HT, after stroke related to AF, has not been established, yet the decision to initiate the anticoagulant is particularly relevant in this context.

The aims of the present analysis of the early initiation of direct anticoagulation after stroke in patients with atrial fibrillation (EIDASAF) study were (1) to evaluate the incidence and the characteristics of early HT in patients with acute ischemic stroke related to AF and (2) to investigate the effect of the presence and severity of WMD on the development of early HT.

## 2 METHOD

Data included in the present study were derived from the database of the EIDASAF study (D'Anna et al., 2020). EIDASAF was an observational, retrospective, single‐center study based on consecutive patient data collected from 2010 to 2017 from the Hyper Acute Stroke Unit of Imperial College London collected. Patients with recent (<4 weeks) ischemic stroke and known or newly diagnosed AF were enrolled into the study. The EIDASAF study was approved by the Health Research Authority (HRA) for collection of data within the NHS. The research did not require review by the UK HRA's Research Ethics Service as it fell into the category of "Research limited to use of previously collected, nonidentifiable information." Informed consent was not a legal requirement as the research was carried out using data collected as part of routine care and any researchers outside of the patients’ direct care team only had access to pseudoanonymized data.

On admission, stroke severity was assessed in all patients using the National Institutes of Health Stroke Scale (NIHSS). A cranial computed tomography (CT) without contrast or brain magnetic resonance imaging (MRI) was performed on admission in all patients as part of routine clinical care to exclude intracranial hemorrhage. Acute reperfusion therapies with tissue plasminogen activator (tPA) and/or mechanical thrombectomy (MT) were delivered as per standard local protocol, when appropriate. Stroke unit care, monitoring and treatment were provided according to current international recommendations for acute ischemic stroke. Attending physicians made all the decisions regarding the timing of initiation of anticoagulant treatment.

**FIGURE 1:**
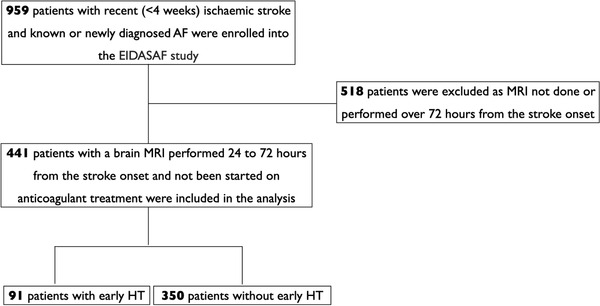
Study flow chart. AF, atrial fibrillation; MRI, magnetic resonance imaging; EIDASAF, early initiation of direct anticoagulation after stroke in patients with atrial fibrillation study; HT, hemorrhagic transformation

For the purpose of the present analysis, we considered only patients who had a brain MRI performed 24–72 h from their stroke onset and who had not been started on anticoagulant treatment (Figure 1) in line with previous studies (Paciaroni et al., 2018, 2008). The brain MRIs were performed with a 1.5T or 3T MRI machine (Siemens AG, Munich, Germany), following an institutional protocol for acute stroke. This protocol included diffusion‐weighted imaging (DWI) at B1000 and apparent diffusion coefficient (ADC) sequences, a fluid attenuated inversion recovery (FLAIR) sequence, a T1, T2 weighted and gradient echo (GRE) sequences (T2‐MRI). The following MRI brain variables were analyzed: (1) stroke volume on DWI, (2) subcortical white matter hyperintensity (i.e., leukoaraiosis) rated according to the Fazekas scale (Del Zoppo et al., 1992) and (3) presence of HT. Stroke lesion volumes were delineated manually on DWI sequences on axial slices using MRIcroN (version 12/2009) (Wahlund et al., 2001). The program automatically calculated the infarct volume in milliliter. The Fazekas classification system is a scale ranging from 0 (no WMD) to 3 (high WMD) (Fazekas et al., 1993). HT was defined as hypointensity on GRE sequence, within the infarcted area or outside the infarct zone, corresponding with iso‐ or hypointensity on T1‐weighted and iso‐or hyperintense signals on T2‐weighted/fluid attenuation recovery images detected only on brain MRI performed during hospitalization. HT was categorized into hemorrhagic infarction (HI) or parenchymal hemorrhage (PH) according to the European cooperative acute stroke study (ECASS) classification (Del Zoppo et al., 1992). HI was defined as small petechiae along the margins of the infarct (HI‐1) or as more confluent petechiae within the infarcted area but without space‐occupying effect (HI‐2). PH was defined as hematoma in <30% of the infarcted area with some slight space‐occupying effect (PH‐1) or as dense hematoma ≥30% of the infarcted area with substantial space‐occupying effect or as any hemorrhagic lesion outside the infarcted area (PH‐2). In case of more than one hemorrhagic lesion on brain scan, the worst possible HT category was assumed. HT was considered symptomatic if it was not seen on the admission brain scan and there was, subsequently, either a suspicion of hemorrhage or a decline in neurological status (an increase of ≥4 points in NIHSS). These MRI brain scans were inspected and rated by an experienced stroke researcher (MM) who was blinded to clinical information. The study analysis began after the stroke researcher reached high reliability (i.e., intraclass correlation analysis >0.90) on a single researcher's repeat assessments of 50 randomly selected brain scans.

Data on known stroke risk factors were collected as follows: age, sex, current cigarette smoking, history of hypertension (blood pressure > 140/90 mm Hg at least twice before acute stroke or already under treatment with antihypertensive drugs), history of treated but uncontrolled hypertension (use of antihypertensive medication but ≥140/90 mm Hg), history of diabetes mellitus (fasting glucose level >126 mg/dl pre‐prandial on two examinations, glucose level >200 mg/dl postprandial, or HbA1c > 6.5% or under antidiabetic treatment), hyperlipidemia (total cholesterol > 200 mg/dl or triglyceride > 140 mg/dl or already on lipid‐lowering therapy), history of symptomatic ischemic heart disease (myocardial infarction, history of angina or previous diagnosis of multiple lesions on thallium heart isotope scan or evidence of coronary disease on coronary angiography), history of symptomatic peripheral arterial disease (intermittent claudication of presumed atherosclerotic origin; or ankle/arm systolic blood pressure ratio <0.85 in either leg at rest or history of intermittent claudication with previous leg amputation, reconstructive surgery or angioplasty), previous stroke/ transient ischemic attack and previous intracranial hemorrhage (ICH). The prescription of any antiplatelet or anticoagulant before admission and the use of these agents on admission and during the follow‐up period were recorded. The CHA2DS2VASc score (2 points for history of stroke or age older than 75 years and 1 point each for congestive heart failure, hypertension, diabetes mellitus, vascular disease, age between 65 and 74 years and female sex) was calculated after the index event. Systolic and diastolic blood pressure and blood tests including hemoglobin level, platelet count, eGFR and creatinine were captured on admission. The HAS‐BLED score (1 point each for hypertension, abnormal renal and liver function, stroke, bleeding, labile INR, elderly, drugs or alcohol) was calculated after the index event.

### 2.1 Statistical analysis

Continuous variables are presented as mean with standard deviation (SD) if values are normally distributed or as median with interquartile range (IQR) when they do not follow the normal distribution. We compared the distribution of continuous variables between groups with *t*‐test or Wilcoxon rank‐sum test as appropriate, whereas categorical values were compared with chi‐square tests. A multivariable logistic regression model was fitted with HT as the outcome and age, DWI lesion volume, NIHSS, tPA, MT, previous ICH and Fazekas scale score as independent variables. We fitted two variations of the model, with Fazekas scale score as a continuous variable in one and as a categorical variable in the other. Similar regression models were also fitted using HI and PH as outcomes and the same set of independent variables. Statistical significance was set at 0.05. All the analyses were conducted with Stata 15.1 (StataCorp, College Station, TX).

## 3 RESULTS

Note that 441 patients were included in the analysis (study flow chart in Figure 1). Ninety‐one patients (20.6%) had any HT of which 68 (15.4%) were HI and 23 (5.2%) were PH. Eight patients had a symptomatic HT (two in the patients with HI and six in the patients with PH). The demographics and clinical characteristics of patients with and without HT are shown in Table 1. Patients were similar in both groups but patients with HT more frequently had suffered an intracranial hemorrhage previously (*p* = .009) and less frequently had peripheral vascular disease (*p* = .027).

**TABLE 1:** Demographics and clinical characteristics of patients with and without hemorrhagic transformation (HT)

	HT (*n* = 91)	No HT (*n* = 350)	*p* value
Age, year (mean, SD)	76.8 (10.3)	77.1 (11.7)	.786
Male, *n* (%)	52 (57.1%)	182 (52.0%)	.548
Smoking, *n* (%)	9 (11.4%)	33 (12.5%)	.864
Hypertension, *n* (%)	68 (74.7%)	259 (74.0%)	.888
Diabetes, *n* (%)	25 (27.5%)	75 (21.4%)	.220
Hypercholesterolemia, *n* (%)	50 (55.0%)	170 (48.6%)	.279
Coronary artery disease, *n* (%)	20 (22.0%)	83 (23.7%)	.727
Heart failure, *n* (%)	7 (7.7%)	35 (10.0%)	.504
Peripheral vascular disease, *n* (%)	2 (2.2%)	32 (9.1%)	.027
Previous ischemic stroke, *n* (%)	21 (23.1%)	75 (21.4%)	.734
Previous TIA, *n* (%)	11 (12.1%)	43 (12.3%)	.959
Previous ICH, *n* (%)	5 (5.5%)	4 (1.1%)	.009
Previously known AF, *n* (%)	66 (72.5%)	264 (75.4%)	.570
NIHSS on admission (mean, SD)	7.16 (6.05)	5.98 (6.20)	.126
Use of anticoagulant before the admission, *n* (%)	23 (25.3%)	128 (36.6%)	.043
Use of antiplatelets before the admission, *n* (%)	33 (36.3%)	101 (28.9%)	.171
tPA, *n* (%)	14 (15.4%)	49 (14.0%)	.737
MT, *n* (%)	3 (3.3%)	12 (3.4%)	.951
CHA2DS2VASC (median, IQR)	5 (4–6)	5 (4–6)	.887
Systolic blood pressure on admission (median, IQR)	148.5 (134–165)	149 (130–169.5)	.643
Diastolic blood pressure on admission (median, IQR)	82 (70–92)	80 (70–94)	.887
Hb g/L, (median, IQR)	129 (92–137)	127 (89–141)	.846
Platelet, ml x 10^–3^, (median, IQR)	220 (177–265)	222 (185.5–271.5)	.896
Platelet, ml x 10^–3^ < 150, *n* (%)	11 (13.6%)	38 (12.0%)	.704
eGFR, ml/min, (median, IQR)	67 (49–80)	66 (50–78)	.377
Creatinine (median, IQR)	82 (71.5–104.5)	84 (70–107)	.806

Abbreviations: AF, atrial fibrillation; HT, hemorrhagic transformation; ICH, intracranial hemorrhage; IQR, interquartile range; MT, mechanical thrombectomy; NIHSS, National Institutes of Health Stroke Scale; SD, standard deviation; TIA, transient ischemic attack; tPA, tissue plasminogen activator.

In terms of neuroimaging findings, patients with HT had a larger infarct volume compared to patients without HT (*p* < .001) (Table 2). There was also a statistically significant difference in the distribution of the Fazekas score according to the four different classes in the two cohorts (*p* = .001) (Table 2).

**TABLE 2:** Magnetic resonance imaging (MRI) characteristics of patients with and without hemorrhagic transformation (HT)

	HT (*n* = 91)	No HT (*n* = 350)	*p* Value
DWI lesion volume, ml (median, IQR)	11.43 (2.54–43.92)	0.6 (0–4.01)	<.001
Fazekas scale score, *n* (%)			<.001
Fazekas scale score 0	8 (8.8%)	143 (40.9%)	
Fazekas scale score 1	27 (29.7%)	79 (22.6%)	
Fazekas scale score 2	30 (33.0%)	94 (26.9%)	
Fazekas scale score 3	26 (28.6%)	34 (9.7%)	

Abbreviations: DWI, diffusion‐weighted imaging; HT, hemorrhagic transformation; IQR, interquartile range.

The distribution of the Fazekas score across the four different classes differed significantly when we compared patients without HT to patients with HI and PH (*p* < .001) (Table 3). Notably, all these patients with PH showed a WMD severity score of 3 according to the Fazekas classification system.

**TABLE 3:** Magnetic resonance imaging (MRI) characteristics of patients without hemorrhagic transformation (HT), with hemorrhagic infarction (HI) and parenchymal hemorrhage (PH)

	No HT (*n* = 350)	HI (*n* = 68)	PH (*n* = 23)	*p* Value
DWI lesion volume, ml (median, IQR)	0.6 (0–4.01)	12.42 (2.89–45.12)	5.04 (2.52–38.53)	<.001
Fazekas scale score, n (%)				<.001
Fazekas scale score 0	143 (40.9%)	8 (11.8%)	–	
Fazekas scale score 1	79 (22.6%)	27 (39.7%)	–	
Fazekas scale score 2	94 (26.9%)	30 (44.1%)	–	
Fazekas scale score 3	34 (9.7%)	3 (4.4%)	23 (100%)	

Abbreviations: HT, hemorrhagic transformation; HI, hemorrhagic infarction, for analysis purpose we considered HI‐1 and HI‐2 together (HI); PH, parenchymal hematoma, for analysis purpose we considered PH‐1 and PH‐2 together (PH); IQR, interquartile range.

### 3.1 Hemorrhagic transformation and timing of oral anticoagulants

After the index acute stroke, 72.0% (252/350) of the patients without HT initiated oral anticoagulant therapy, whereas 67.0% (61/91) of patients with HT were treated (*p* = .352) during the hyper acute stroke unit (HASU) inpatient stay. The median time (days) to initiation of oral anticoagulant therapy was substantially longer in patients with HT (6.5; IQR 3.5–13) compared to patients without HT (3; IQR 2–8) (*p* = .001).

Among patients with HT, 49 of 68 (72.1%) with HI were anticoagulated, while 12 of 23 (52.2%) of patients with PH initiated oral anticoagulant therapy after the index acute stroke (*p* = .125) during the HASU inpatient stay. Patients with HI (6; IQR 4–14) and PH (8; IQR 3–13) showed a longer median time from stroke onset until initiation of oral anticoagulant therapy compared to patients without HT (*p* < .001).

### 3.2 Independent predictors of early HT

On multivariable analysis (Table 4), HT was independently associated with higher infarct volume (OR, 1.03; 95% CI, 1.01–1.05; *p* < .001), higher Fazekas scores (OR, 1.94; 95% CI, 1.47–2.57; *p* < .001) and history of previous ICH (OR, 4.80; 95% CI, 1.11–20.80; *p* = .036).

**TABLE 4:** Multivariable model: Predictive factors for hemorrhagic transformation (HT)

Variable	Odds ratio	95% CI	*p* Value
Age (years)	1.01	0.98–1.03	.935
DWI lesion volume (ml)	1.03	1.01–1.05	<.001
NIHSS	1.01	0.96–1.06	.767
Fazekas scale score	1.94	1.47–2.57	<.001
tPA	1.14	0.53–2.47	.726
MT	0.42	0.70–2.52	.342
Previous ICH	4.80	1.11–20.80	.036
Age (years)	1.01	0.98–1.03	.812
DWI lesion volume (ml)	1.03	1.02–1.04	<.001
NIHSS	1.01	0.96–1.07	.706
Fazekas scale score			
0	1.00 (ref.)	–	–
1	5.11	2.05–12.70	<.001
2	4.38	1.74–11.01	.002
3	12.09	4.48–32.65	<.001
tPA	1.11	0.51–2.42	.783
MT	0.34	0.53–2.15	.286
Previous ICH	5.03	1.11–22.82	.036

Abbreviations: HT, hemorrhagic transformation; ICH, intracranial hemorrhage; MT, mechanical thrombectomy; NIHSS, National Institutes of Health Stroke Scale; tPA, Tissue plasminogen activator.

In the model where the Fazekas score was included as a categorical variable (Fazekas scale score 1, 2, 3), Fazekas score of 1 (OR, 5.11; 95% CI, 2.05–12.70; *p* < .001), Fazekas score of 2 (OR, 4.38; 95% CI, 11.74–11.01; *p* = .002) and Fazekas score of 3 (OR, 12.09; 95% CI, 4.48–32.65; *p* < .001) were significantly associated with HT in patients with acute stroke and AF.

On multivariable analysis (Table 5), HI was positively associated with higher infarct volume (OR, 1.03; 95% CI, 1.02–1.04; *p* < .001), Fazekas score of 1 (OR, 5.02; 95% CI, 2.02–12.48; *p* = .001), Fazekas score of 2 (OR, 4.36; 95% CI, 1.74–10.97; *p* = .002) and history of previous ICH (OR, 7.53; 95% CI, 1.37–41.21; *p* = .020). We did not find any statistically significant independent predictors of PH (Table 6). Of note, Fazekas score and MT did not show any variation within PH categories; hence, they were automatically excluded from the respective regression model.

**TABLE 5:** Multivariable model: Predictive factors for hemorrhagic infarction (HI)

Variable	Odds ratio	95% CI	*p* value
Age (years)	0.99	0.97–1.02	.654
DWI lesion volume (ml)	1.03	1.02–1.04	<.001
NIHSS	0.99	0.94–1.05	.789
Fazekas scale score	1.34	0.98–1.83	.070
tPA	1.24	0.55–2.78	.602
MT	0.56	0.89–3.47	.530
Previous ICH	4.57	0.94–22.28	.060
Age (years)	1.00	0.97–1.02	.835
DWI lesion volume (ml)	1.03	1.02–1.04	<.001
NIHSS	1.01	0.95–1.07	.812
Fazekas scale score			
0	1.00 (ref.)	–	–
1	5.02	2.02–12.48	.001
2	4.36	1.74–10.97	.002
3	1.01	0.18–5.65	.987
tPA	1.08	0.48–2.45	.854
MT	0.47	0.67–3.29	.446
Previous ICH	7.53	1.37–41.21	.020

Abbreviations: HI, hemorrhagic infarction, for analysis purpose we considered HI‐1 and HI‐2 together (HI); ICH, intracranial hemorrhage; MT, mechanical thrombectomy; NIHSS, National Institutes of Health Stroke Scale; tPA, tissue plasminogen activator.

**TABLE 6:** Multivariable model: Predictive factors for parenchymal hematoma (PH)

Variable	Odds ratio	95% CI	*p* Value
Age (years)	1.02	0.93–1.12	.672
DWI lesion volume (ml)	1.04	1.00–1.08	.047
NIHSS	1.06	0.90–1.24	.514
Fazekas scale score	–	–	–
tPA	0.84	0.85–8.23	.877
MT	–	–	–
Previous ICH	3.11	0.20–48.17	.416

Abbreviations: ICH, intracranial hemorrhage; MT, mechanical thrombectomy; NIHSS, National Institutes of Health Stroke Scale; PH: parenchymal hematoma, for analysis purpose we considered PH‐1 and PH‐2 together (PH); tPA: Tissue plasminogen activator,.

## 4 DISCUSSION

The main finding of our analysis is that the presence and severity of WMD is associated with an increased risk of development of early HT.

In previous studies, the relationship between WMD and risk of HT was controversial. Paciaroni et al. (2018) did not find a statistically significant association between WMD and HT in patients with acute stroke and AF assessed with a second CT scan or MRI performed 24–72 h from stroke onset. In their study, leukoaraiosis was only dichotomized into absent versus present (Wahlund et al., 2001). A retrospective study of 122 consecutive stroke patients who underwent MRI of the brain 6–60 h after stroke onset did not find that the extent of chronic white matter lesions, classified according to the Fazekas criteria, differed between patients with or without HT (Kablau et al., 2011). Similarly, Pande et al. (2020) did not show a statistically significant difference in terms of presence of microvascular changes in patients with and without HT. In both studies, the majority of strokes were classified as noncardioembolic. Conversely, another retrospective study of 207 patients presenting with acute stroke and AF and/or rheumatic heart disease evaluated with brain MRI or CT scan up to 7 days after admission supported a potential role for leukoaraiosis in predicting HT after acute ischemic stroke (Wei et al., 2019). Moreover, Bivard et al. (2016) found an association between small vessel disease and increased risk of HT in 229 patients with ischemic stroke. However, 127 patients (55.5%) had been treated with intravenous thrombolysis before the follow‐up scan and only 59 patients (25.8%) had cardioembolic stroke. Despite the limitation of our retrospective study design, our study strongly suggests that WMD has an effect on the frequency and severity of HT in cardioembolic stroke. Compared to previous studies, our patient cohort was rather large and homogenous, and it was limited to AF‐related stroke. Moreover, all included patients underwent a brain MRI 24–72 h from the stroke onset and before the initiation of the anticoagulant treatment.

In our study, 20.6% of participants had any HT (HI 15.4; PH 5.2%). In previous studies, the proportion of stroke patients with HT varied largely between 50.81% (Wei et al., 2019) and 5.9% (Valentino et al., 2017) in the literature. Difference in the time interval between stroke onset, in the modality of follow‐up imaging and in the population of stroke patients included in the studies partially explains the discrepancies between the reported different rates. MRI, as used in our study, is more sensitive than CT for detection of blood components, especially the deoxyhemoglobin and hemosiderin, after acute ischemic stroke (Arnould et al., 2004; Renou et al., 2010).

Similar to previous studies, the volume of the brain infarct (Butcher et al., 2010; Tong et al., 2001) was associated with an increased risk of HT in patients with acute ischemic stroke and AF. The 2016 ESC Guidelines (Kirchhof et al., 2016) for initiation of anticoagulation after ischemic stroke suggest to initiate anticoagulation on the basis of the severity NIHSS score (NIHSS < 8: mild stroke; NIHSS 8–15: moderate stroke; NIHSS ≥ 16: severe stroke). The interdisciplinary expert opinion of the European Heart and Rhythm Association (Heidbuchel et al., 2013) recommends starting direct oral anticoagulants after stroke depending on the infarct size. Future research is needed to provide reliable radiological predictors of the risk of HT in cardioembolic stroke to better characterize the cohort of patients who may benefit from early initiation of anticoagulation.

Our study has several limitations. The reported associations in our nonrandomized study could undoubtedly be influenced by numerous potential confounders even though adjusted statistical models were used to reduce them. The retrospective nature of data collection is another limitation of the study. The participants in this study were all ischemic stroke patients with AF who represent a specific population and may limit the generalizability of our findings. Finally, a long‐term follow up was not available for analysis. The strengths of our study include the sample size and the homogeneity of the subjects

In conclusion, the presence and severity of WMD was associated with an increased risk of early development of early HT in patients with acute stroke and AF. Our findings provide additional information to predict the occurrence of HT and as consequence they enable safer initiation of anticoagulation therapy, even during the acute phase of cardioembolic stroke. Further evidence is needed to provide reliable radiological predictors of the risk of HT, in cardioembolic stroke, to better characterize the cohort of patients who may benefit from early initiation of anticoagulation.

## CONFLICT OF INTEREST

Lucio D'Anna, Filippos T Filippidis, Kirsten Harvey, Marilena Marinescu and Paul Bentley report no disclosures.

Eleni Korompoki: Speaker Honoraria: Amgen, INNOVIS, Pfizer; Advisory boards: Pfizer. Roland Veltkamp has received received fees for consulting and speaker honoraria from Bayer, Boehringer Ingelheim, BMS, Pfizer, Daich Sankyo, Portola, Biogen, Medtronic, Morphosys, Amgen as well as research support from Bayer, Boehringer, BMS, Pfizer, Daiichi Sankyo, Medtronic, Biogen outside of the present work.

### PEER REVIEW

The peer review history for this article is available at https://publons.com/publon/10.1002/brb3.2250.

## Data Availability

The data that support the findings of this study are available on request from the corresponding author. The data are not publicly available due to privacy or ethical restrictions.
